# Exposure to parental and sibling smoking and future intentions to smoke among 13-15 years old school age children in Saudi Arabia

**DOI:** 10.11604/pamj.2021.38.158.27825

**Published:** 2021-02-12

**Authors:** Sudhakar Vundavalli, Ahmed Ali Alfawzan, Vardharajula Venkata Ramaiah, Moataz Alruwaithi, AbdulMajeed AlMogbel, Merin Mathew

**Affiliations:** 1 Department of Preventive Dentistry, College of Dentistry, Jouf University, Sakakah, Saudi Arabia,; 2 Department of Preventive Dentistry, College of Dentistry, Qassim University, Al Rass, Saudi Arabia,; 3 Department of Dental Hygiene, College of Applied Health Sciences, Qassim University, Al Rass, Saudi Arabia,; 4 Eastern Riyadh Specialized Dental Center, Ministry Of Health, Riyadh, Saudi Arabia,; 5 Department of Orthodontics and Pediatric Dentistry, College of Dentistry, Qassim University, Qassim, Saudi Arabia,; 6 Department of Prosthetic Dental Sciences, Dental Materials Division, College of Dentistry, Jouf University, Sakakah, Saudi Arabia

**Keywords:** Risk Indicators, smoking, siblings, school children, Saudi Arabia

## Abstract

**Introduction:**

prevalence of smoking in school children is alarming in Saudi Arabia and little is well-known about the aspects stimulating such behaviours in secondary school children. The aim of this study was to assess the association between influence of parent/sibling/peer smoking and future intentions to initiate smoke among 13-15 years old school children in Al Ras town, Saudi Arabia.

**Methods:**

a cross sectional survey was conducted in Al Ras city during first quarter of 2019. Data was collected from 492 secondary school children who were selected through multistage stratified cluster sampling. Pretested, self-administered Arabic questionnaire was used to collect data about socio-demographic and prevalence of current smoking behaviours and associated factors. Descriptive statistics was done initially, following by binomial regression to assess the predictors of current smoking and future smoking intentions.

**Results:**

the overall prevalence of smoking in respondents was 22.7% and statistically significant difference in smoking prevalence's between boys and girls was observed (40% vs 5.6%). Among the predictors of current smoking, smoking habits in siblings and getting pocket money over 200 Saudi riyals are found to be significant. Siblings smoking (odds ratio: 6.4) and poor academic performance (odds ratio: 3.2) were the two most important factors influencing children's intentions of smoking.

**Conclusion:**

smoking prevalence in secondary school children of Al Ras was similar to national data. Since, influence of siblings, getting more pocket money and poor academic performance were found to important predictors of children smoking behaviours and attitudes, health education programs should address these factors to be effective.

## Introduction

Saudi Arabia stands at a better place in terms of health indicators among Gulf cooperation countries (GCC) and life style related health problems were leading causes of mortality like any developed nations [1]. Smoking is considered as one of the life style related factor. Approximately one lakh young people globally start smoking on any given day [2]. Various studies in the past reported about the prevalence of smoking in Saudi children, Al-Zalabani and Kasim reported 15% prevalence of smoking in 11-19 years old students in Madinah [3] and peer influence was found as significant predictor for smoking in these students, another study reported by Abdelrahim in secondary school children found that prevalence of smoking was 17.3% and pooracademic performance and peer influence were found to be independent predictors for smoking [4].

Smoking is considered as potential risk factor for several health problems such as oro-pharyngeal cancer, lung cancer, stroke, cardiovascular disease and levies a momentous economic and social burden on families and society [5]. Therefore, prevention of smoking at primordial or primary level is one of the major public health goals for many countries. Strategies or efforts to delay or prevent children or young adults to start smoking gained much public health importance, because the earlier the age of initiation of smoking, difficult to quit the habit and pose health problems at earlier age [6]. Secondary school children who are about to enter adulthood represents an important age cohort in which health related habits and attitudes more likely to be shaped during that age. Such children are an important target group, as regular smoking habit is not yet developed in majority of such children [6].

Although some of the children among the participants are not current smokers, they may have developed future intentions to initiate smoking [7]. Future intentions to smoke will help in predicting subsequent smoking behavior as per theory of planned behavior [8]. Sequentially, various factors such as attitudes, family and peer influence, social desirability etc will influence the intentions to smoke. There is a dearth in literature about such factors in Saudi children which influence the children´s intention to smoke and such knowledge can help to develop a comprehensive smoking prevention program. Therefore, the present study aimed to assess the association between social factors (parent/sibling/friend smoking) and future intentions to smoke among 13-15-year-old school children in Al Ras city, Saudi Arabia (KSA).

## Methods

**Design:** a cross sectional study

**Source of data:** secondary school children aged between 13-15 years who are native residents of Al Ras were selected for this study. Multi stage stratified cluster sampling was used to select the participants. In the initial phase, schools in Al Ras were divided into two strata (male vs female), from each stratum two schools were selected by simple random method and the children in the selected schools were selected through systematic random sampling.

**Inclusion criteria:** children aged between 13-15-year-old; children who gave oral consent and also got written consent for participation from their parents/legal guardian; only Saudi nationals were included in this study.

**Exclusion criteria:** children who are medically compromised/any medical condition which may adversely affect outcome variables; foreign nationals were not be included in original sample; child who failed to give/obtain consent to be part of this study.

**Sample size:** sample size was calculated based on the prevalence of smoking in secondary school children (15-18%) reported in various previous studies among the secondary children [3,9,10]. Considering 95% CI and 80% power, 472 children weresufficient to detect significant difference of 10% with design effect of two. So, total sample size was increased to 500, anticipating 20-25% chances of non-participation. Sample size was calculated using the below mentioned formula:

$$N=\frac{\left(z\alpha +z\beta )P(1-P\right)}{d^{2}}$$

hence, final sample size of the study was 500 secondary school children.

**Data collection:** data was collected using a pretested closed ended questionnaire. Items in the questionnaire was developed as per guidelines mentioned by World Health Organization [11] and other previously used and validated questionnaire in Arabic language [12]. Apart from demographic information of the participants, 36 questions were included in the questionnaire and final version of the questionnaire was verified by two public health experts who are well versed with both English and Arabic. Internal consistency of the final questionnaire was tested with Crohbach´s Alpha, which was found to be excellent (0.96) and test-retest reliability was assessed with correlation coefficient (0.86).

Initially, permission from the school authorities was obtained and children were informed about this questionnaire study and parental consent form was given to children who agreed to participate. After one week of initial visit, classroom based survey was conducted for the children who got parental consent and participants were asked to fill the anonymous questionnaire and return it back on the same day before leaving school.

**Study variables:** one of the outcome measures, smoking initiation is considered as any change in the never smoker status to ever or current cigarette smoker and a student was recorded as never smoker if she/he had never smoked partial or full cigarette or took not even a puff of smoking cigarette. Intention to start smoking has been defined as any future desire to start cigarette smoking.

**Data management:** all relevant data collected during the study was entered into Microsoft Excel. Anonymity was maintained by not recording the name of the participant and each participant will have a serial number. Neither the names of the school nor name of the participants were retrieved from main data, thus confidentiality was maintained.

**Ethical approval and confidentiality:** ethical approval for study protocol was obtained from ethical committee of Qassim University with reference number DRC/007FA/19. Confidentiality of the data was maintained and participants information will not be disclosed without their/parents´ consent.

**Statistical analysis:** collected data was initially entered into Microsoft Excel (Microsoft Windows 10) and analyzed using Statistical Package for Social Sciences (SPSS version 20, Armonk, US). Descriptive statistics was performed initially and independent samples 't' test and Chi-square tests were used for bivariate analysis. Binary logistic regression was used to assess the predictors of cigarette smoking and intentions to start smoking. For all comparisons, P-value ≤0.05 was considered as statistically significant.

## Results

Final data included responses from 492 students out of 500 with response rate of 98.4% and there is no statistically significant gender difference in participation. Table 1 describes the overall prevalence of the smoking among the participants was 22.7% and the majority of the smokers were boys (98 out of 112). Parents´ education status is inversely proportional to the prevalence of smoking in children and more smokers among the children who got pocket money more than 200 Saudi riyals per month. Nearly 30% of the participant children started at before the age of 13, 37.5% stated at the age of 14 and 32.2% of them started at the age of 15 which is statistically not significant (P=0.776).

**Table 1:** comparison of smoking prevalence with basic characteristics

	Total	Current smokers N (%)	Non-smokers N (%)	P value
**Age (in years)**				
13	152	24 (15.7%)	128 (84.3%)	
14	154	40 (30%)	114 (70%)	0.342
15	186	48 (25.8%)	138 (74.2%)	
Overall	492	112 (22.7%)	380 (77.3%)	
**Gender**				
Boys	245	98 (40%)	147 (60%)	
Girls	248	14 (5.6%)	234 (94.4%)	0.001*
Overall	492	112 (22.7%)	380 (77.3%)	
**Father education**				
High school or less	349	71 (20.3%)	278 (79.7%)	0.082
Professional	143	41 (27.9%)	102 (72.1%)	
**Mother education**				
High school or less	374	88 (23.5%)	286 (76.5%)	0.662
Professional	118	24 (20.3%)	94 (79.7%)	
**Pocket money per month**				
<200 Saudi riyals per month	119	28 (23.5%)	91 (76.5%)	0.021*
>200 Saudi riyals per month	373	84 (22.5%)	289 (77.5%)	
**Age at which smoking initiated (in years)**			**Not applicable**	
≤13		34 (30.3%)		0.766
14		42 (37.5%)		
15		36 (32.2%)		

Various factors associated with smoking were compared in Table 2. Sixty two percent of the smokers has one of the parent as a smoker in contrast to non-smoking group in which it is just 31.8% and 8% of both parents in smoking group and 16.1% in non-smoking group having both parents are smokers, which is statistically not significant (0.18). Of siblings in smoking group, 92.1% had the habit of smoking in contrast to 19.5% in non-smoking group siblings which is statistically significant (0.002). Of the children in smoking group, 43.8% reported that one or more of their friends were smokers against 27.4% of them in non-smokers group which is statistically significant (0.001). Among the attitudes related questions statistically significant difference was found for the statement “safe to smoke year or two” and 81.2% of smokers and 32.6% of non-smokers has this attitude (Table 2).

**Table 2:** association of few smoking related factors with smoking

	Smokers	Non smokers	P-value
	112	380	
**Parents smoking**			
No	34 (30.3%)	198 (52.1%)	0.18
One parent	70 (62.5%)	121 (31.8%)	
Both	8 (07.2%)	61 (16.1%)	
**Siblings smoking**			
No	11 (09.8%)	306 (80.5%)	0.02*
Yes (one or more)	101 (91.2%)	74 (19.5%)	
**Friends smoking**			
No	63 (56.2%)	276 (72.6%)	0.001*
Yes (one or more)	49 (43.8%)	104 (27.4%)	
**Attitudes towards smoking**			
Smokers has more friends	92 (82.1%)	231 (60.7%)	0.26
It is important to socialize	85 (75.8%)	236 (62.1%)	0.12
Safe to smoke year or two	91 (81.2%)	124 (32.6%)	0.01*
Difficult to quit once started	83 (74.1%)	289 (76%)	0.62
Effects sports performance	45 (40.1%)	125 (32.8%)	0.08
Makes you gain or lose weight	38 (33.9%)	138 (36.3%)	0.44

In Table 3, comparisons were made for various factors among the students who has the intention to start smoking and students who do not have intentions to start smoking. Intention to smoking perceptions are more with the students who are unhappy with academic performance (32.6%) at school compared to students who are happy about their academic performance (13%) which is statistically significant. Students who have siblings (one or more) as smokers has more intentions to smoke (75.6%) compared to students who has less percentage of smoker siblings (10.4%) which is statistically significant (0.001). Among the attitude related factors, the attitude of “it is important to socialize” is predominant factor associated with intentions of smoking among the participants.

**Table 3:** association of intentions to start smoking with various smoking related factors among non-smoking children

	Intention to smoke	No intention to smoke	Total	P-value
**Gender**				
Boys	56 (38%)	91 (62%)	147	0.718
Girls	32 (13.6%)	202 (86.4%)	234	
Overall	88 (23.1%)	293 (76.9%)	380	
**Age (in years)**				
13	13 (10%)	115 (90%)	128	0.316
14	28 (24.4%)	86 (75.6%)	114	
15	48 (34.7%)	90 (65.3%)	138	
**School performance**				
Happy with school performance	24 (13%)	164 (87%)	184	0.042*
Unhappy with school performance	64 (32.6%)	132 (67.4%)	196	
**Parents smoking**				
No	34 (17.1%)	164 (82.9%)	198	0.236
One parent	31 (25.6%)	90 (74.4%)	121	
Both	13 (21.3%)	48 (79.7%)	61	
**Siblings smoking**				
No	32 (10.4%)	274 (89.6%)	306	0.001*
Yes (one or more)	56 (75.6%)	18 (24.4%)	74	
**Friends smoking**				
No	58 (21%)	218 (79%)	276	0.456
Yes (one or more)	30 (28.8%)	74 (71.2%)	104	
**Attitudes towards smoking**				
Smokers has more friends	88 (38%)	143 (62%)	231	0.282
It is important to socialize	88 (37.2%)	148 (62.8%)	236	0.012*
Safe to smoke year or two	88 (71%)	36 (29%)	124	0.892
Difficult to quit once started	88 (30.4%)	201 (69.6%)	289	0.564
Effects sports performance	88 (70.4%)	37 (29.6%)	125	0.064
Makes you gain or lose weight	88 (63.7%)	50 (36.3%)	138	0.920
**Pocket money per month**				
<200 Saudi riyals per month	18 (19.7%)	73 (80.3%)	91	0.282
>200 Saudi riyals per month	60 (20.7%)	229 (79.3%)	289	

Logistic regression was done with dependent variable as current smoker or non-smoker and independent variables, which were found to be significant in bivariate analysis, were included in the model (Table 1 and Table 2). Among the predictors, students who receive more than 200 Saudi riyals per month as pocket money has 2.4 times more chances of being current smoker and students who have one or more smoker siblings has 6.7 times more chances of being smoker which is statistically significant (P=0.001) (Table 4).

**Table 4:** predictors of smoking: binary logistic regression

	OR	95% CI	P-value
**Gender**			
Boys	1.11	0.60-1.95	0.23
Girls	0.96	0.54-1.66	0.48
**Pocket money per month**			
<200 Saudi riyals per month	0.68	0.38-1.16	0.34
>200 Saudi riyals per month	2.4	0.98-3.6	0.01*
**Siblings smoking**			
No	0.92	0.62-1.32	0.63
Yes (one or more)	6.7	4.20-7.31	0.001*
**Friends smoking**			
No	1.09	0.53-2.18	0.82
Yes (one or more)	1.70	0.92-3.14	0.06
**Attitude**			
Safe to smoke year or two	0.92	0.56-1.47	0.73

Another logistic regression model was performed to identify of the predictors of “intentions of smoking” among non-smoker students (Table 5). Among the independent variables, students who are not happy with academic performance at school will have 3.2 times more intention of start smoking which is statistically significant (P=0.02) and students who have one or more smoker siblings are 6.4 times more risk of intention to start smoking in future which is statistically significant (P=0.000).

**Table 5:** predictors of intention to start smoking: binary logistic regression

	OR	95% CI	P-value
**School performance**			
Happy with school performance	0.82	0.47-1.48	0.55
Unhappy with school performance	3.2	1.82-4.24	0.02*
**Siblings smoking**			
No	0.77	0.42-1.30	0.35
Yes (one or more)	6.4	4.81-7.54	0.000*
**Friends smoking**			
No	0.94	0.54-1.76	0.86
Yes (one or more)	1.76	0.94-3.22	0.07
**Attitude**			
It is important to socialize	1.28	0.89-1.84	0.18

## Discussion

The key purpose of current study is to assess the parental and siblings influence on future intention of smoking in 13-15 year old school children and other associated factors that make them vulnerable to initiate smoking behavior in Al Ras, Saudi Arabia. The results of this study suggest that smoking habit in siblings and friends along with poor performance in school influence the students´ future intentions to smoke. Parents influence on smoking was a predominant cognitive factor influenced children´s smoking behaviors and intention from studies reported years back [13,14], while siblings influence and peer pressure becoming increasingly more significant in this decade. However, in this study no clear associations were detected between parents smoking behaviours and children´s intentions to start smoking which is similar to study reported by Al-Zalabani *et al*. in which friends and siblings has more influence on smoking behaviors of children [3]. This can be explained by smoking is more common in the fathers rather than mothers, more over polygamy is common in Saudi Arabia which further makes Saudi children to spend most of their family time with mothers and siblings and their influence is more on them compared to father. However, further research may be needed to assess thepossible mechanisms involved in the role of parents on secondary school children´s cognitive susceptibility towards cigarette smoking.

The findings in this study suggest that around three-fourth of non-smoking children who said that they have the intention of smoking has one or more siblings as smokers and influence of friends is less in this study compared to other studies reported earlier [12,15]. This influence of siblings can be ascribed to multiple factors. Firstly, while young children in the early school years are more probable to spend much of time with their mother/father and siblings and it is the older children that spends more time with friends. So, siblings predominantly signify children's prime social environment, and are more likely to be additional proximal stimuli on children´s exposure to initiate smoking other than the father/mother [16]. Second, sibling and friends smoking behaviour is probably be the less explicit than parent smoking and this influence the children´s perception of treating smoking as exciting or cool and a social desirability [16]. Considering all together, the results indicate that sibling smoking behaviours is one of the major contributors to secondary school children's cognitive behaviour to start smoking. However, more evidence is necessary and further research is desirable to explore the exact mechanisms involved in siblings influence to design smoking cessation or prevention programs.

Current study findings suggest that smoking prevalence is more in the boys than in girls (5.6% vs 40%) and the overall prevalence of smoking in this age group was 22.7% which is less compare to national survey conducted by ministry of health (MOH) in 2013 [17]. However, in logistic regression model gender is not significantly associated with smoking behaviour. Results suggest that girls has less intention to smoke than boys (13.6% vs 38%) which is similar to studies reported by McGee *et al*. [18] in which girls has less smoking intentions compare to boys in England and another study reported by Karma Mc Kelvey *et al*. [19] from Jordan about intention of starting smoking in 12-14 year old school children in which male children had 2.5 more times intention to start smoking compared to female counterparts. Again, influence of gender on intention to start smoking was not statistically significant in bivariate analysis and gender was not included in list of predictors of smoking intentions in logistic regression model.

The finding that children who are receiving pocket money more than 200 SAR had 2.4 more chances of being current smoker compare to children who received less than 200 SAR per month (Table 4). These findings are similar to the study reported by Al-Zalabani *et al*. [3] from Madinah in which statistically significant difference in smoking prevalence among the children who received more than 300 SAR and children who receive less than 300 SAR per month. This explains about the indirect role of parents in providing unmonitored pocket money to children and further emphasizes the efficiency of existing law that selling of products to children below 18 years [16].

Academic performance in the school was another significant factor examined in this study, children who are unhappy about their academic performance in school has 3.2 times more intention to start smoking compared to children who are happy about academic performance (Table 5). These findings are similar to study about substance abuse among high school seniors by Brittany AB *et al*. in which children with perceived poor academic performance has 2.7 times more chances of substance abuse [20] and another study reported by Mumtaz *et al*. in that low academic performance was one considered as one of the important predictors of smoking in Saudi adolescents [21]. To our knowledge, only one other study reported by Al Nohair *et al*. [22] has concurrently examined the role of academic performance in secondary and intermediates students and most important predictor of smoking was (odds ratio=5.2) poor academic performance among the students [23]. Future prospective studies probing the impact of the immediate social atmosphere in these children are necessary.

Encouragingly, nearly three-fourth of children demonstrated their towards resilience towards non-smoking and this study encompasses the smoking research literature in preteen children by exploring the role of parents, friends and siblings on their cognitive liability to smoking initiation among Saudi children. However, this study has few limitations. First, the study design is cross sectional in nature, therefore the exact casual relationships cannot be recognized. In addition, this study just assessed the current intentions of smoking which may or may not be changed at later age. However, several previous researches have demonstrated that factors such as intention to smoke in early ages are predictive of future adult smoking behaviors [16,24]. The second limitation of the study was self-reported nature of the data collection tool, which creates a probability that the responses can be over or under reported as influenced by recall bias or social desirability. Nevertheless, it is difficult to conduct any interventional studies or longitudinal studies because of ethical issues. Moreover, past literature has demonstrated that children can provide reliable data about their habits and perceptions provided that confidentiality is assured for them [25,26].

## Conclusion

From the findings of this study, it can be concluded that siblings influence, poor academic performance and getting more pocket money are important predictors of secondary school children´s current smoking behaviours and intention to start smoking. Individual smoking cessations programs should be complemented with group health education methods to mitigate the adverse influences of immediate social environment on children´s smoking behaviours.

### What is known about this topic

- Parental influence on children´s smoking initiation is well known fact;
- Peer pressure encourages smoking initiation.

### What this study adds

- Siblings play a major role in children´s intentions to smoke;
- Academic performance and getting more pocket money were also found to be motivators of children´s smoking initiation.
